# 3D Single-Virus Tracking: Advances in Methodology and Labeling Strategies Towards Probing the Virus–Epithelium Interaction

**DOI:** 10.3390/v18050521

**Published:** 2026-04-30

**Authors:** Yuxin Lin, Haoting Lin, Donggeng Yu, Kevin Welsher

**Affiliations:** Department of Chemistry, Duke University, Durham, NC 27708, USA; yuxin.lin@duke.edu (Y.L.); haoting.lin@duke.edu (H.L.); shawn_yudg@163.com (D.Y.)

**Keywords:** epithelium, real-time 3D single-virus tracking, active feedback tracking, early-stage viral infection

## Abstract

The epithelium represents the first line of defense against viral infection, yet the precise mechanisms by which viruses penetrate this complex barrier remain incompletely understood. Single-virus tracking (SVT) has emerged as a powerful fluorescence microscopy approach to directly visualize viral dynamics with nanometer spatial precision and millisecond temporal resolution. In this review, we survey recent progress in SVT methodologies, from image-based approaches to active feedback techniques, and assess their capacity to resolve viral behavior in physiologically relevant epithelial models. We further evaluate advances in virus labeling strategies—including fluorescent proteins, organic dyes, and nanoparticles—that enable prolonged observation while preserving infectivity. By integrating developments in optical instrumentation and molecular labeling, SVT is increasingly capable of capturing critical processes, including extracellular diffusion, receptor engagement, internalization, and trans-epithelial transport. Finally, we discuss current challenges, including limited penetration depth, photobleaching, and the complexity of 3D epithelial tissues, and outline future opportunities to extend SVT towards in situ and tissue-level studies. Together, these advances position SVT as a transformative tool to illuminate virus–epithelium interactions and guide therapeutic strategies.

## 1. Introduction

### 1.1. Epithelium: The Frontier Against Viral Infection

A virus is a submicron infectious pathogen consisting of a nucleic acid genome (DNA or RNA), capsid, and often a lipid envelope decorated with proteins responsible for cellular binding [1]. Viral proteins are among the most essential components of viruses, which typically exist in both the capsid and envelope. Viral proteins not only protect the enclosed genome from degradation but also enable recognition of host cells during initial binding and virus-to-cell fusion [2]. As such, these proteins are frequent targets for therapeutics and viral labeling. Viruses can invade the human body by sexual contact, blood-to-blood transmission, the fecal–oral route, from mother to fetus, and through the respiratory tract [3]. The human body has developed immune defenses against viral invasion by both specific and nonspecific mechanisms [4]. Studies have confirmed that nonspecific defenses respond more quickly than specific defenses and limit most viral infections in the early stages, typically in bodily fluids and the epithelium [5,6].

**Figure 1:**
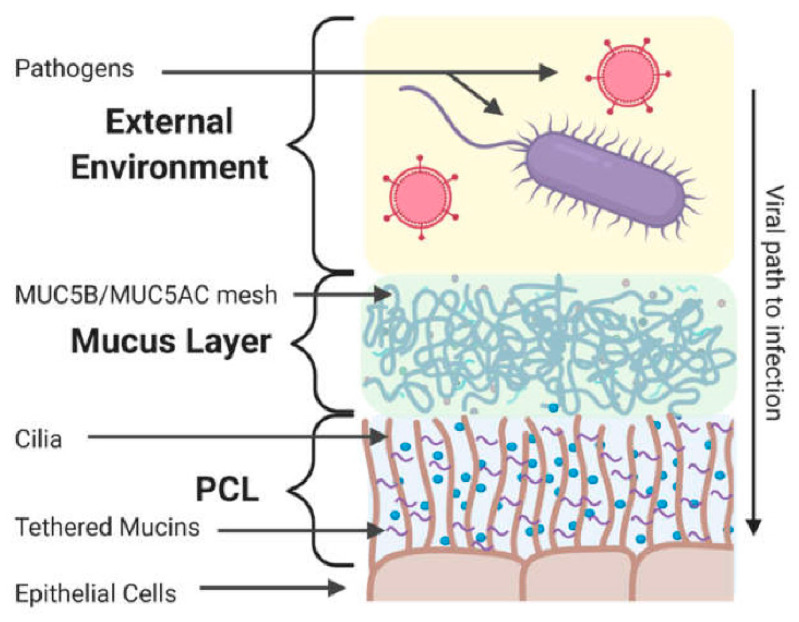
Cross-section of the respiratory tract mucosal barrier. Epithelial cells of the proximal airways are protected by a dense PCL and an overlying mucus gel layer. Pathogens can be slowed or trapped in this restrictive gel while the coordinated beating of underlying ciliated epithelial cells propels this gel away from the more vulnerable distal airways towards gastrointestinal clearance. Reprinted from Ref. [9] under the terms of CC BY 4.0.

The epithelium is a complex tissue with an extended mucus layer and forms the first mechanical, chemical, and immunological barrier against exogenous threats, including viral infection [7]. For example, the airway epithelia act as a dense trap for respiratory viruses [8]. Before a viral infection can be initiated, viruses must penetrate the extracellular barrier, including the mucus layer and the periciliary layer (PCL) [9] (Figure 1). The mucus layer is an integral component of mucosal epithelial cells, which forms the barrier between the external environment and the host epithelium. The mucus is composed of heavily sialylated glycoproteins aptly named mucins, which some pathogens use to bind to host tissue [10]. Epithelial cells also release receptor-rich compartments into the extracellular space, which act as decoys to invading virions [11]. The PCL, comprising a 5–8 μm thick mesh of cilia- and microvilli-bound mucins, is a size-excluding barrier to particles larger than 40 nm in diameter [12]. Ideally, the functional and healthy epithelial barrier should prevent most viral penetration and infection of the underlining epithelium. Unfortunately, viruses have evolved various strategies to facilitate their entry and infection [13]. Understanding the interactions that enable pathogens to cross this complex barrier is critical to treating a wide range of diseases, including the recent SARS-CoV-2 pandemic. The optimal approach to studying viral penetration through the epithelia is through real-time direct visualization of infection from the perspective of a single virion, with sufficient spatiotemporal resolution to uncover critical molecular detail. Tracking single viruses as they pass through the epithelia would present an ideal methodology to study this critical step in the infection process.

### 1.2. Single-Virus Tracking Overview

Single-particle tracking (SPT) is a quantitative microscopy method that measures the dynamics of individual particles in their native environment [14]. Since the first application of SPT in live cells [15], researchers have been applying this technique to study the cell–particle interaction by capturing phenomena that cannot be extracted from bulk experiments [16]. While various imaging modalities can be employed for SPT, fluorescence microscopy is widely used for its high sensitivity, versatility, viability, and compatibility with live-cell imaging [17,18]. Being an essential subcategory of SPT, single-virus tracking (SVT) has successfully unraveled many fundamental biological secrets dating back to the 1980s [19,20], including extracellular viral diffusion [21,22], virus–receptor interaction [23,24,25], virus internalization [26,27,28], intracellular trafficking [29,30], genome release [31], assembly and egress of viruses [32,33], and cell-to-cell transmission [34].

Based on fundamentally different particle localization philosophies, modern SVT methods can be roughly categorized into image-based SVT and active feedback SVT. Typically, image-based SVT methods illuminate a fixed field of view and sequentially take a series of diffraction-limited images with detectors, usually cameras. Afterwards, localizations are obtained from the intensity-weighted centroid of the image of the virus. Trajectories are then constructed by connecting positions serially frame-by-frame. For these image-based methods, the temporal resolution of trajectories is limited by the imaging rate, which is primarily determined by the camera readout time and the required exposure time to achieve a desired signal-to-noise ratio. These limitations in the temporal resolution are exacerbated when extending to three dimensions. In image-based SVT, the axial information is typically obtained by stepping through different focal planes and reconstructing a 3D volume, resulting in at least an order of magnitude reduction in the temporal resolution. As an alternative, strategies have been developed to engineer the point spread function (PSF) to encode axial information in a two-dimensional image. The benefits and trade-offs of such an approach are discussed in Section 2.4 below.

In contrast to conventional image-based methods, active feedback SVT methods narrow down the field of view to exclusively focus on a single particle. The particle’s position is estimated in real time and fed into a feedback loop to effectively hold the particle stationary in the focal volume of the microscope, typically using either a piezoelectric nanopositioner or galvo scanning mirror [35]. The readout from the repositioning device represents the particle’s real-time position. In these methods, the major limitations in temporal resolution are the response time of the repositioning hardware and the photon detection rate. While active feedback tracking requires a narrow field of view to achieve real-time and high spatiotemporal resolution single-virus tracking, recent work has shown that multi-resolution approaches can recover the critical cellular context, which is necessary to understand the interaction between viral particles and cells.

### 1.3. Challenges of Single-Virus Tracking in Epithelium

Despite the central role of the epithelium as the first barrier to viral infection, direct applications of single-virus tracking in epithelial systems remain relatively scarce [21,36]. This limited body of work is not due to a lack of biological importance but rather reflects the substantial technical challenges associated with resolving viral dynamics in physiologically relevant epithelial environments. Some of the fundamental challenges to extracting dynamic molecular-scale information from viral trajectories include the requirement of high spatiotemporal resolution to quantify infection dynamics, the lack of long-term viability of cells and fluorescent labeling, and the cellular complexity and complex geometry of the epithelium.

**Figure 2:**
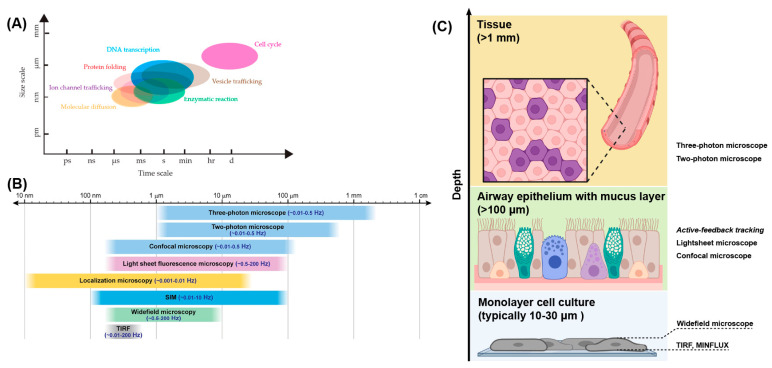
(**A**) The temporal and spatial scales of biological processes. The time windows and size windows are approximate, and each process has its own situation-dependent scale. Reprinted from Ref. [56] under the terms of CC BY 4.0. (**B**) Typical fluorescence microscopy methods used in living samples over indicated spatiotemporal scales. Note that boundaries between methods are “fuzzy” and only approximate. Reprinted from Ref. [57] under the terms of CC BY 4.0. (**C**) Typical cell models used to study epithelium. Some applicable fluorescence microscopy methods with enough penetration are listed next to the corresponding models.

The crucial steps in viral infection cover a vast range of spatial and temporal scales. A complete viral infection cycle lasts from hours to days at the macro or tissue level, while critical molecular-scale steps, such as viral attachment and receptor binding, may happen on the millisecond timescale or faster [37,38] (Figure 2A). Given the limited total number of photons that can be emitted by fluorophores, particularly organic dyes and fluorescent proteins, it is very challenging to observe a single viral particle with high spatiotemporal resolution over a sufficient time period to capture the entire infection process. To address this challenge, advanced virus labeling techniques have been developed to increase the brightness and photostability, which will be discussed in Section 4. The speed with which viral particles move is another complicating factor in capturing the entire infection process. The diffusion coefficient, a typical measurement of how fast particles diffuse, of viruses in different stages of infection varies dramatically. A recent study has revealed the average diffusion coefficient for VSV-G VLPs traveling in the extracellular matrix in an epithelium mimicking model is 1.29 ± 0.44 μm^2^/s, which is at least two orders of magnitude higher than bound or internalized particles [21], indicating that viral particles can remain highly mobile in biologically relevant extracellular environments. The viral diffusion coefficient in the extracellular environment or buffered solutions can reach up to ~10 μm^2^/s, with the actual number dictated by the size and environment of the particles. Particles diffusing at these high speeds are difficult or impossible to capture by conventional methods.

The thick and complex environment of the epithelium not only functions as a natural barrier against external pathogens but also presents significant obstacles to observing virus–epithelium interactions in situ or in live tissue [39]. Some commonly used cell models to study the epithelium include 2D monolayer epithelial cells [40], air–liquid interface cultures [41], organoids [42,43,44], spheroids [45,46], organ-on-a-chip [47], and 3D bioprinting [48,49]. Among them, 2D cell models of the epithelium are the simplest and most cost-effective [50]. However, these 2D models have still shown a loss of biological relevance when compared with native environments due to flatter cellular morphology, monolayer structure, exposure to culturing media, lack of cell junctions, poor differentiation, and unnatural proliferation [51,52,53,54]. To mimic and observe native virus–epithelium interactions, more complex models are needed.

More complex models of the epithelium present barriers to live-cell microscopy in general, and to SVT in particular. Observations must be made through deeper, more complex, and highly scattering environments. The total thickness of 3D cell models can range from hundreds of micrometers to even several centimeters [43,44,49], which easily exceeds the penetration depth of most existing fluorescent microscopy methods (Figure 2B,C). The working range of microscopy methods is not the only challenge. As the thickness increases in 3D cell models, the heterogeneity in refractive index introduces massive light scattering, which leads to a loss in collected fluorescent signal, as well as a spread in the PSF. Both these factors limit the ability to track small signals at depth. Similarly, multiple layers of cells, even without fluorescent labeling, result in massive autofluorescence from biomolecules, including FAD and NADH, which will negatively affect the signal-to-noise ratio and make trajectories unreliable or unobservable [55].

Due to the challenges listed above, the dynamics of virions in the epithelial space have been poorly explored, limiting our collective knowledge of particle behavior to isolated and surface-bound cells. To demystify the virus–epithelium interaction, an ideal microscopy method should have nanometer-level spatial resolution in three dimensions, millisecond-level temporal resolution, enough penetration depth (over 100 μm) to cooperate with complex biological models from the airway epithelium all the way to tissue samples, and a large working z-range (tens of micrometers at least) to capture events that occur over different focal planes.

In this review, we will first survey existing SVT methods, from image-based to active feedback approaches. This review covers several methods critical to the development and evolution of single-particle tracking, and they are discussed in terms of their potential application to SVT. Each method discussed will be evaluated in terms of its potential to study the virus–epithelium interaction. Emerging virus labeling techniques, which facilitate single-virus tracking, will be discussed in the context of advancing SVT into more challenging environments. Finally, the future possibilities and challenges of applying SVT to the epithelium are discussed.

## 2. Image-Based Single-Virus Tracking Methods

Since the emergence of SVT, image-based methods have seen the greatest application across different fields due to their intuitive mechanism and the availability of commercial instrumentation. In image-based SVT, images of viruses in live−cell environments are acquired sequentially in a rigid field of view. Particle locations are identified in each image or frame and concatenated to form trajectories. Ideally, multiple particles within a single field of view can be tracked simultaneously.

In this section, we will discuss traditional microscopies and some emerging advances in image-based tracking methods. Their applications in SVT will be highlighted along with the potential challenges translating into epithelia.

### 2.1. Wide-Field Microscopy

The simplest setup for SVT is wide-field or epifluorescence microscopy. As suggested by the method’s name, a light source excites a wide field of view, and all fluorescent signals, both in- and out-of-focus, are indiscriminately collected by an objective lens with a high numerical aperture and detected with a camera [58]. Owing to its large depth of field and simple implementation, epifluorescence microscopy can be readily applied for 2D SVT in simple samples [16]. For example, Abidine et al. tracked the 2D diffusion of herpes simplex virus-1 (HSV-1) in live cells and proposed different functions of host glycocalyx components upon initial HSV–cell interactions [59]. Recent developments have also pushed the temporal and spatial limits of wide-field microscopy. For instance, SpeedyTrack exploits the rapid charge-shifting capability of EM-CCDs to encode temporal information spatially, enabling microsecond-resolution single-molecule tracking and super-resolution mapping in the wide field without hardware modification [60].

However, as both in- and out-of-focus signals from the sample are captured, traditional wide-field microscopy cannot extract 3D information and is not suitable for thick and highly scattering samples. Another noticeable drawback is that wide-field microscopy excites all focal planes simultaneously, and the unnecessary excitation leads to more rapid photobleaching compared to more modern microscopies.

### 2.2. Total Internal Reflection Fluorescence Microscopy

**Figure 3:**
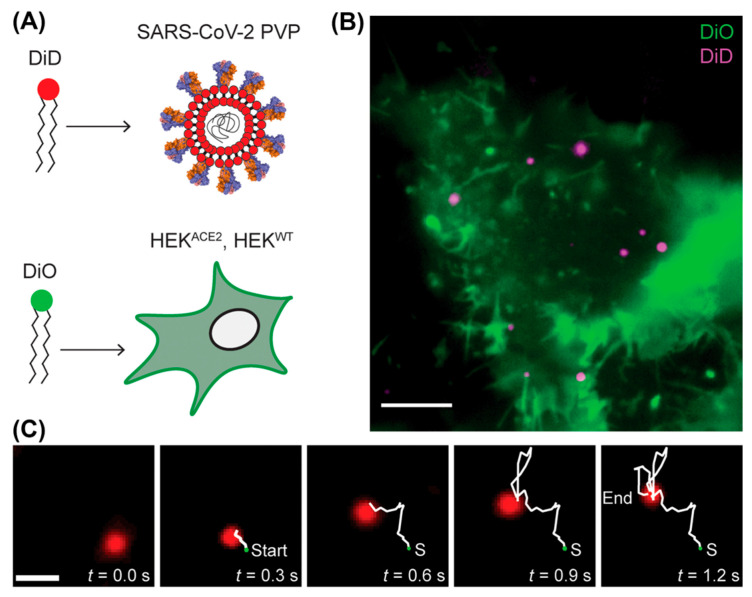
An example of single-virus tracking with TIRF microscopy. (**A**) Pseudotyped viral particles (PVPs) are labeled with DiD (red), and HEK cells are labeled with DiO (green) to observe the lipid envelope/membrane. (**B**) Example TIRF image of DiO-labeled HEK cells with DiD-labeled PVPs. Scale bar, 5 μm. (**C**) An example PVP trajectory. Scale bar, 1 μm. Reprinted with permission from Ref. [25] under the terms of the Creative Commons CC BY license.

In 1981, Daniel Axelrod developed total internal reflection fluorescence (TIRF) microscopy [61]. In this method, an evanescent wave is generated near the liquid–glass interface with a prism or an objective lens with a numerical aperture above 1.4. The non-propagating nature of the evanescent wave eliminates the excitation of fluorophores away from the surface, which increases the signal-to-noise ratio and sensitivity while minimizing photobleaching and photodamage. TIRF is a powerful tool for investigating virus–cell interactions near the coverslip surface. For instance, Christie et al. benefited from the high spatiotemporal resolution of TIRF and tracked the interaction of DiD-labeled pseudotyped viral particles (PVPs) with different SARS-CoV-2 spike protein variants and DiO-labeled target cells (hACE2 receptor-negative wild-type HEK cells and HEK cells stably expressing hACE2) to learn how mutation influences infectivity [25] (Figure 3). TIRF also favors SVT on thin artificial membrane systems, where membrane components can be easily adjusted and kinetic information on virus–membrane interactions can be extracted. For example, Peerboom et al. performed single HSV tracking on a biomimetic surface modified with glycosaminoglycans (GAGs) to study the lateral diffusion of HSV due to multivalent virus–GAG interactions [62].

Unfortunately, as the intensity of the evanescent wave decays exponentially with the distance from the interface, the penetration depth of TIRF is limited to below 200 nm [63]. Even with optical modifications, methodologies such as highly inclined and laminated optical sheet (HILO) microscopy remain limited to simple monolayer cell cultures. This restriction to surface (or near-surface) observation makes TIRF and its cousins unsuitable for tracking viruses in 3D epithelial models [64].

### 2.3. Confocal Microscopy

With the aim of imaging through deep brain tissues, confocal microscopy was first patented by Marvin Minsky in 1961, but it was not experimentally achieved until about 30 years later [65,66,67]. The basic concept is to achieve point illumination and block out-of-focus photons from reaching the detector using two pinholes, one each on the excitation and detection beam paths. Currently, there are two major implementations of confocal microscopy: laser scanning confocal microscopy (LSCM) and spinning disk confocal microscopy (SDCM). Both techniques can be applied to perform SVT.

LSCM scans one pixel at a time, and the entire field of view is imaged by either moving the entire sample with a motorized stage or, more commonly, by scanning the combined excitation and detection paths with galvo or resonant scanning mirrors. As a sophisticated and highly commercialized method, LSCM is widely used to study the viral infection process. A recent LSCM-SVT study directly observed viral diffusion through a synthetic mucus gel network [68]. Rich information was extracted from viral trajectories, including diffusion coefficient, degree of confinement, pore size, and virus dissociation constant. These trajectories suggested that the mobility of influenza A virions can be limited physically and biochemically by both the pore size and virus–receptor interactions [68]. Using LSCM in monolayer epithelial cells, another study was able to probe the earliest stage of respiratory syncytial virus infection and revealed that viral entry occurred through lipid rafts and was highly dependent on the kinase Pak-1 [69].

Due to its ability to generate 3D images, a moderate penetration depth of about 100 μm, and ease of integration with other techniques, LSCM has been favored by researchers across various fields. However, to apply a microscopy method to the virus–epithelium interaction, high spatiotemporal resolution across a large axial range is critical. The frame rate of LSCM is fundamentally limited by the available photons in each pixel and requires a Z-stack to acquire 3D information. Given a modest 512 × 512-pixel lateral resolution, 16 Z-slices over an axial range, and a scanning rate of 1 μsec/pixel, it will take over 4 s to image the whole volume, resulting in poor temporal resolution for a 3D-SVT method. Shrinking down the imaging volume could increase the temporal resolution. However, the observation duration will suffer as the probability of the virus escaping the imaging range is higher. Thus, LSCM is not a suitable method to trace highly dynamic viral particles, especially in complex 3D environments.

SDCM is specially designed to increase the imaging rate by simultaneously exciting multiple points in the same focal plane with a Nipkow disk [70]. The result is a dramatic increase in the imaging frame rate as compared to LSCM, though the scaling issues still remain when applied to a three-dimensional sample. SDCM is a ubiquitous tool in studies of the virus–cell interaction. Ma et al. tracked pseudo-SARS-CoV-2 virus-like particles, dual-labeled on the membrane with a lipophilic dye (DiO) and viral protein R (Vpr) fused with mCherry, in different 2D human respiratory cell models and visualized the entry process at the single-particle level [71]. According to the authors, SDCM imaging was performed at 10 s intervals in 2D to avoid photobleaching. In another example, Real et al. performed live-cell imaging on mucosal epithelium reconstructed in vitro and visualized the trans-epithelial movement of HIV-1 via transcytosis [72]. In this example, 3D live-cell imaging was accomplished with a multiphoton scanning laser over hundreds of micrometers in depth, while 3D trajectories of fluorescent viruses were analyzed for a maximum of 20 μm along the Z coordinate, with minutes-scale temporal resolution.

SDCM is faster than LSCM for acquiring 3D images. However, the two methods still share some weaknesses. These include diffraction-limited spatial resolution, limited imaging depth with one-photon excitation, and photobleaching [73]. The pinhole does provide optical sectioning; however, the small excitation area and short dwell time require high excitation power, leading to bleaching of fluorophores, local heating, and photodamage to the cell. Researchers typically have to sacrifice temporal resolution to reduce photobleaching and phototoxicity when using LSCM and SDCM in live-cell experiments.

### 2.4. Point Spread Function Engineering

**Figure 4:**
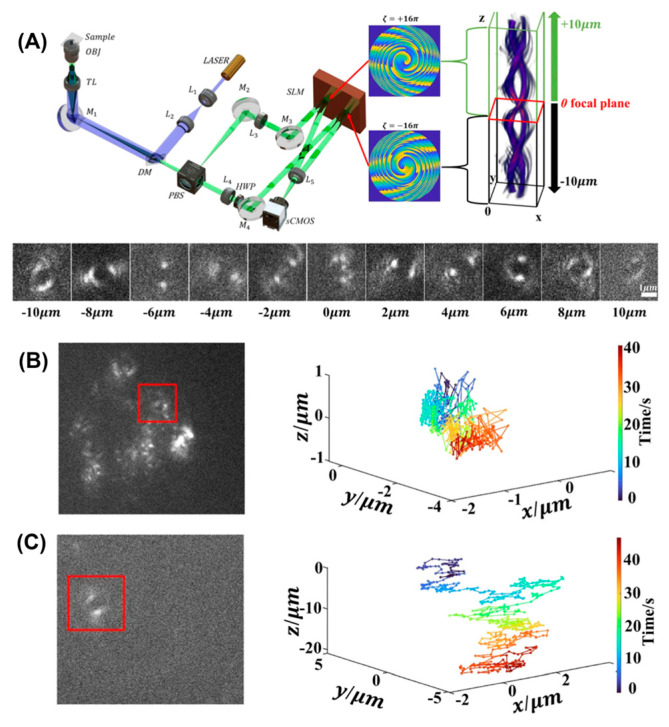
An example of SPT methods based on PSF engineering. (**A**) Optical setup and 3D stereograms of two defocused 2*π*-DH-PSF combinations. (**B**) Three-dimensional trajectory of fluorescent microspheres in HeLa cells. (**C**) Three-dimensional trajectory of fluorescent microspheres in saliva. Reprinted from Ref. [81] under the terms of CC BY 4.0.

The PSF is the image of a point object and is an important determinant of a microscope’s performance. The standard PSF of a high magnification light microscope is relatively insensitive along the *Z*-axis, and as a result, does not provide much axial information. This is primarily due to the symmetry of the PSF above and below the focal plane, making it possible to determine if an object is out of focus but difficult to determine the direction. Researchers have successfully engineered novel PSF shapes that are extremely sensitive to the axial position of the detected object with high photon efficiency [74]. With the adoption of PSF engineering, the throughput of 3D particle tracking is greatly improved, as a single camera readout now encodes X, Y, and Z position information. Commonly used PSF designs include astigmatism [75], phase-ramp [76], double helix [77], accelerating beam [78], corkscrew [79], and saddle-point PSFs [80]. Unfortunately, most of these have a relatively narrow range of axial localization, generally below 5 μm, and, therefore, are not useful for tracking dynamic particles. By combining different designs, more complicated PSF patterns can offer a larger z-range of up to 20 μm and have been successfully employed to track fluorescent nanospheres [81] (Figure 4). An interesting recent advance by Nozue et al. utilized a birefringent substrate, mica, to mount the sample, with an axial tracking range of up to 30 µm and a simplified optical setup [82]. However, in PSF engineering, the increased Z-range comes with a more complex PSF that spans many pixels in the image plane. This leads to fewer photons in each pixel, having a dramatic effect on the signal-to-noise ratio in the resulting image and poorer localization in trajectories. Furthermore, the particle must be moving slowly enough such that it is stationary within a single camera exposure to avoid motion-induced blur and mislocalization. As a candidate for SVT in the challenging epithelial environment, these methods have not yet been translated to high-background and high-scattering samples. Another drawback is that these methods require wide-field illumination, leading to rapid photobleaching and potentially phototoxicity.

### 2.5. Light-Sheet Microscopy

**Figure 5:**
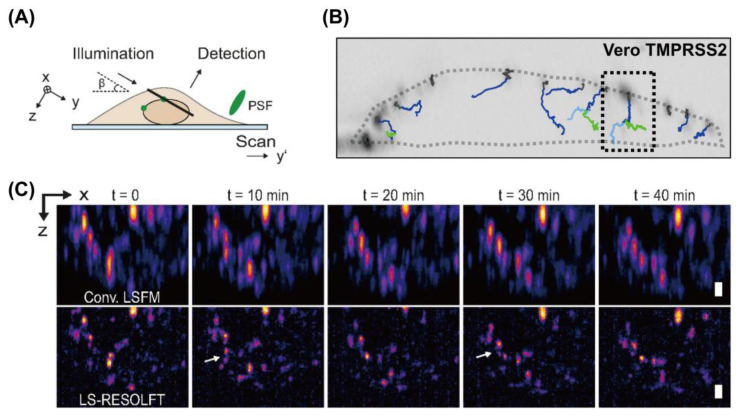
Light-sheet-based single-virus tracking. (**A**) Schematic of light-sheet illumination and fluorescence detection for a single particle. Reprinted from Ref. [90] under CC BY. (**B**) Representative live-cell single-virus trajectories of VSV-SARS-CoV-2 labeled with eGFP internally and Atto 565 on the spike proteins. Traces highlight combined trajectories with colocalized eGFP and Atto 565 signal (blue) or separated signal from eGFP (green) and Atto 565 (light blue). Reprinted from Ref. [87] under CC BY-NC-ND. (**C**) Live-cell light-sheet-RESOLFT imaging of assembly of rsEGFP2-labeled HIV-1 at the HeLa cell surface, shown with a maximum intensity projection. HIV-1 assembly sites resolved by LS-RESOLFT but not by conventional LSFM are highlighted by white arrows. Scale bar: 5 µm. Reprinted from Ref. [90] under CC BY.

As mentioned in Section 2.3, the use of confocal imaging techniques for SVT is limited by slow acquisition speeds, especially for processes involving large volumes. Additionally, although the pinhole blocks fluorescence from out-of-focus objects from reaching the detector, the fluorophores continue to be excited and can undergo photobleaching even when not being directly measured. This makes it challenging to perform long-term volumetric imaging with LSCM. Light-sheet fluorescence microscopy (LSFM) was developed to address shortcomings of LSCM [83]. LSFM uses a different illumination pattern to increase the imaging speed and suppress photobleaching. In LSFM, a thin excitation light sheet (*XY* plane) is generated perpendicular to the optical axis (*Z*-axis) (Figure 5). The result is that a thin slice of the sample is illuminated and detected by a camera, without the need to scan the laser to generate an XY image. Volumes are generated by translating the sample relative to the light sheet or scanning the light sheet in the axial direction. Ideally, particles imaged via LSFM are only illuminated once per volume, providing far superior resistance to photobleaching compared to LSCM [84]. Images and volumes are also acquired dramatically faster with LSFM, as millions of voxels are collected in parallel, with speeds of over 300 volumes per second reported after modification [85]. We note here that these imaging rates are strongly dependent on the axial extent of the volume and the brightness of the objects in the image, as they determine the minimum exposure time that can be used. Overall, the high acquisition speed and low photodamage of LSFM provide great temporal resolution and imaging duration for SVT with contemporaneous imaging of the volumetric environment. Owing to these advantages, lattice light-sheet microscopy (LLSM) with structured illumination has been used to study the intracellular trafficking of both influenza and SARS-CoV-2 virions at imaging speeds of 2 s per stack [86,87].

One factor that affects the application of LSFM to highly scattering samples is occlusion, wherein the plane excitation is blocked by objects within the sample. Scanning from multiple directions has suppressed the side effects of occlusion along the path of the light sheet. Glaser et al. combined the ideas of multidirectional selective plane illumination microscopy (mSPIM) and digital scanned light-sheet microscopy (DLSM) to provide angular diversity in light-sheet imaging and minimize shadowing artifacts [88]. Likewise, Bosse et al. introduced a two-axis system to TIRF microscopy and produced a virtual “ring-sheet” for capturing fast nuclear dynamics of herpesvirus [89]. The rapid rotation of illumination is equivalent to multidirectional excitation and can reduce shading artifacts. Hoyer et al. improved the axial resolution of LSFM via RESOLFT (reversible saturable/switchable optical fluorescence transitions) [90]. This combined method was used to characterize HIV-1 assembly. The challenge of placing two objectives in close proximity has been overcome by coupling a prism to allow them to be placed at an angle greater than 90° or by reducing the number of objectives to one [91]. Bouchard et al. used a scanning mirror to create a moving oblique light sheet [85,92]. The unique scanning–descanning optical configuration allows the use of only one objective lens for light-sheet imaging, opening the possibility of SVT in more complex samples.

Several modifications can be made to conventional LSFM to optimize the technique for tracking viruses deep within scattering samples. Illuminating samples from multiple sides and fusing those images is a simple but effective method to illuminate a larger area than using a light sheet from one direction. An electronic confocal slit can be applied to this design to further suppress the unwanted background from scattered light [93]. Other methods for increasing the penetration depth of LSFM focus on weakening the attenuation of light (by both absorption and scattering) by biological samples using optical sample-clearing techniques [94] and multiphoton excitation [95,96].

### 2.6. Scattering-Based Microscopy

While fluorescence microscopy benefits from the Stokes shift between excitation and emission light, resulting in low background levels, it inevitably suffers from some fundamental limitations. First, particles of interest need to be labeled to be detected. The presence of an exogenous label can trigger artifacts in interactions, where the label itself drives the dynamics rather than the particle or molecule under investigation. Second, even when excited at a saturating light dose, fluorophores have a finite fluorescence emission rate. This limited emission rate restricts the maximum imaging rate and the localization precision. Finally, irreversible photobleaching and phototoxicity limit the total observation time [97]. Scattering-based approaches do not suffer from these obstacles and have emerged as an alternative to fluorescence microscopy. Dark-field microscopy is a type of scattering-based microscopy that can produce high-quality images similar to those achieved with fluorescence staining by detecting scattered light while removing excitation light [98]. However, the intensity of dark-field scattering scales inversely with the square of the object volume, making it challenging to detect single molecules. Interference-based microscopy is a better option for small and weakly scattering objects, as the scattering contrast can be significantly enhanced by introducing a second probe beam to interfere with the scattered light [99]. These methods have been around for a long time but have regained interest due to the limitations of fluorescence-based methodologies.

**Figure 6:**
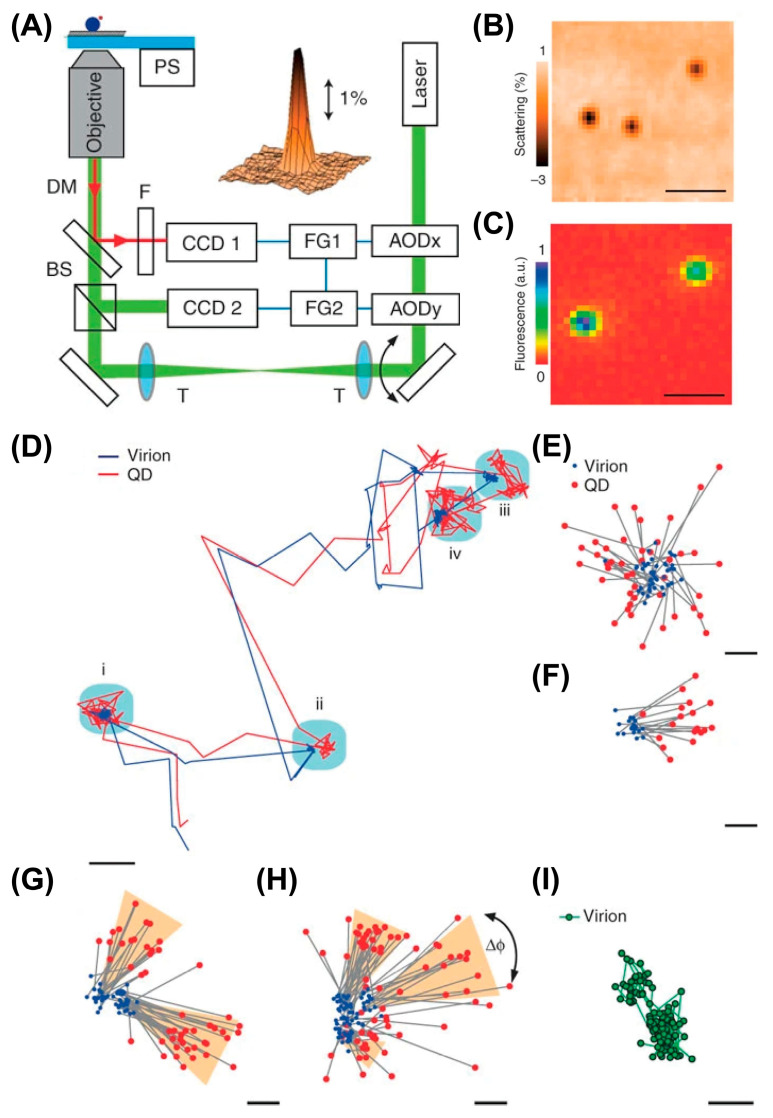
(**A**) Schematic of an iSCAT setup. The surface plot is inverted for clarity. (**B**,**C**) Representative interferometric scattering image of individual SV40 VLPs (**B**) and a corresponding simultaneously acquired fluorescence image (**C**). Scale bars, 1 μm. (**D**) Virion and QD trajectories from 250 consecutive iSCAT imaging frames acquired with an exposure time of 20 ms per image (25 frames s^−1^). Scale bar, 50 nm. (**E**–**H**) Zoom of the highlighted sections i–iv in (**D**). The blue and red dots represent the positions of the virion and the QD, respectively. The gray lines connect corresponding positions across exposures. The orange triangles highlight the confinement of φ. (**I**) iSCAT map of a rocking motion for an unlabeled virion. Scale bars, 10 nm. Reprinted from Ref. [101]. Copyright (2009) Springer Nature.

Although the idea of interferometric imaging can be traced back to a work in 1991 that studied the bending of sliding microtubules using confocal light microscopy in reflection mode [100], interferometric scattering microscopy (iSCAT) as a term and an established method was first introduced in a study of the diffusional dynamics of individual viruses on supported lipid bilayers (Figure 6) [101]. In iSCAT, part of the incident light is reflected at the interface between the glass, while most of it propagates through the interface and interacts with the sample. If the light is coherent, the back-reflected light and the scattered light from the sample interfere with each other and give the detected intensity of the sample as:

(1) $$I_{det}=I_{inc}[r^{2}+{\left|s\right|}^{2}+2r\left|s\right|\cos\varphi ],$$

where $I_{inc}$ is the incident intensity, $r^{2}$ is the reflectivity of the glass–water interface, s is the scattering amplitude of the sample, and φ represents the phase difference between the reflected and scattered fields. As the particle size decreases to the subwavelength range, the pure scattering term ${\left|s\right|}^{2}$ becomes negligible while the interferometric term $2r\left|s\right|\cos\varphi$ scales down much more slowly with respect to particle size and still maintains a detectable signal level, which is dependent on $\sqrt{N}$ (where *N* is the total number of detected photons). Thus, although the interferometric term can be weak at a low signal-to-noise ratio, increasing the incident light and exposure time will still enable the detection of nanoscale objects in the sample. More recent studies have demonstrated the potential of iSCAT microscopy for a wide range of research applications, including single-particle tracking [102], imaging biological nano-objects and their dynamics [103,104,105], and detection and mass measurement of single unlabeled proteins [106,107]. Alternatively, coherent brightfield microscopy detects forward scattering light of nanoparticles and uses transmitted non-scattered light as the reference beam. Coherent brightfield microscopy has been applied for tracking single vaccinia virus particles on a cell membrane with a 100 kHz frame rate [108].

To apply iSCAT to SVT inside living cells, two important challenges need to be addressed. First, in the complex cellular environment, various components strongly scatter light, making it difficult to identify nanoparticles. One solution is to remove the signal from static scatterers and only focus on moving ones, which can be achieved by subtracting or dividing frames. This method, called time-differential iSCAT, was used to track nanosized cargo motion along cytoskeletal filaments [109]. Another approach, similar to confocal fluorescence microscopy, is to modify wide-field iSCAT into confocal iSCAT to reject out-of-focus scattering. This strategy has been applied to track the motion of SARS-CoV-2 colocalized with clathrin-coated pits at the live-cell plasma membrane [110].

Another challenge is that iSCAT typically does not provide direct axial localization of particles, or is limited mainly to those located near the surface, which makes it difficult to fully resolve virus–cell interactions. Although the asymmetry of iSCAT PSF (iPSF) provides some axial information, it is usually limited to a range of a few hundred nanometers [110]. More refined analysis of the iPSF extends the range of iSCAT-based SPT. By establishing correlations between experimental and theoretical radial PSFs, Kasaian et al. were able to track gold nanoparticles of 10–80 nm in diameter diffusing in water, with an axial depth of up to 5 µm [111]. Another approach, conceptually similar to PSF engineering, is to engineer iPSF to be z-sensitive. Huang et al. introduced a double-helix iPSF using a one-dimensional photonic crystal, which enabled SPT over nearly 2 µm axially [112]. Similarly, Brooks et al. introduced a spiral phase iPSF, achieving SPT over an axial range approaching 3 µm [113]. Taken together, these strategies are promising for further applications in SVT within living cells.

## 3. Active Feedback Single-Virus Tracking Methods

While each of the imaging methods above provides numerous benefits to single-virus tracking in live cells, they share a common limitation. The single-virus dynamics that can be read out are limited by the time required to image an entire volume. Even fast imaging methods, such as SDCM or LSFM, are limited in their volumetric imaging rate by the overhead of acquiring images of multiple focal planes and concatenating them into a volume. This limits the temporal resolution of single-particle dynamics to the seconds range at best. Active feedback 3D single-particle tracking methods overcome this imaging overhead to push the temporal resolution to the sub-millisecond level while retaining or even improving the precision of single-particle trajectories. While active feedback tracking methods are varied in their particular application, they share a common general scheme. The position of a particle is measured and used in real time to move the sample or the combined excitation/detection spot to center the particle within the focal volume. While within the focal volume, photons are continuously collected from the moving particle by a sensitive detector, such as a single-photon avalanche diode (SPAD), or an SPAD array, in a recent advance [114]. These photons provide valuable information on molecular dynamics [115,116], fluorophore lifetime to probe local environment [117], and even molecular conformation with Förster resonance energy transfer (FRET) labeling [118,119].

Active feedback single-particle tracking methods are defined by the method in which real-time particle position is measured. Generally, particle localization is achieved by multi-detector modified detection or via patterned excitation [56]. The particles’ deviation from the center is calculated from the estimated real-time position and used in a feedback loop to drive a repositioning device (piezoelectric stage or galvanometer scanning mirror) to lock the dynamically moving particle at the center of the detection volume. For a well-centered particle, the readout from the repositioning device indicates the particle’s real-time position. The temporal resolution becomes limited only by the photon detection rate, easily achieving msec or better localization time. Active feedback methods have the built-in advantage of capturing processes over large axial ranges with no loss of temporal resolution, limited only by the range of the feedback response element or the optical penetration depth.

However, the enhancement of the spatiotemporal resolution of active feedback tracking microscopy originated from converting the large imaging field of view into a narrow scan pattern to optimize the tracking of a single target. As a result, active feedback methods have highly limited throughput, typically only tracking one particle at a time, and have no contextualization for cellular environments when applying to SVT.

In this section, we will first discuss active feedback methods with modified detection. Then, we will discuss methods with engineered excitation. Their application in SVT will be highlighted, along with contextualization methods of the cellular environment.

### 3.1. Active Feedback Single-Virus Tracking with Modified Detection

Active feedback tracking methods with modified detection split the emission signal onto multiple point detectors (SPC-APDs) to monitor corresponding changes in the X, Y, and Z positions of the particle [115]. The signal difference in detectors is employed to calculate the particles’ real-time position, which is then used to drive a feedback element to reposition the particle to the center of the detection volume.

**Figure 7:**
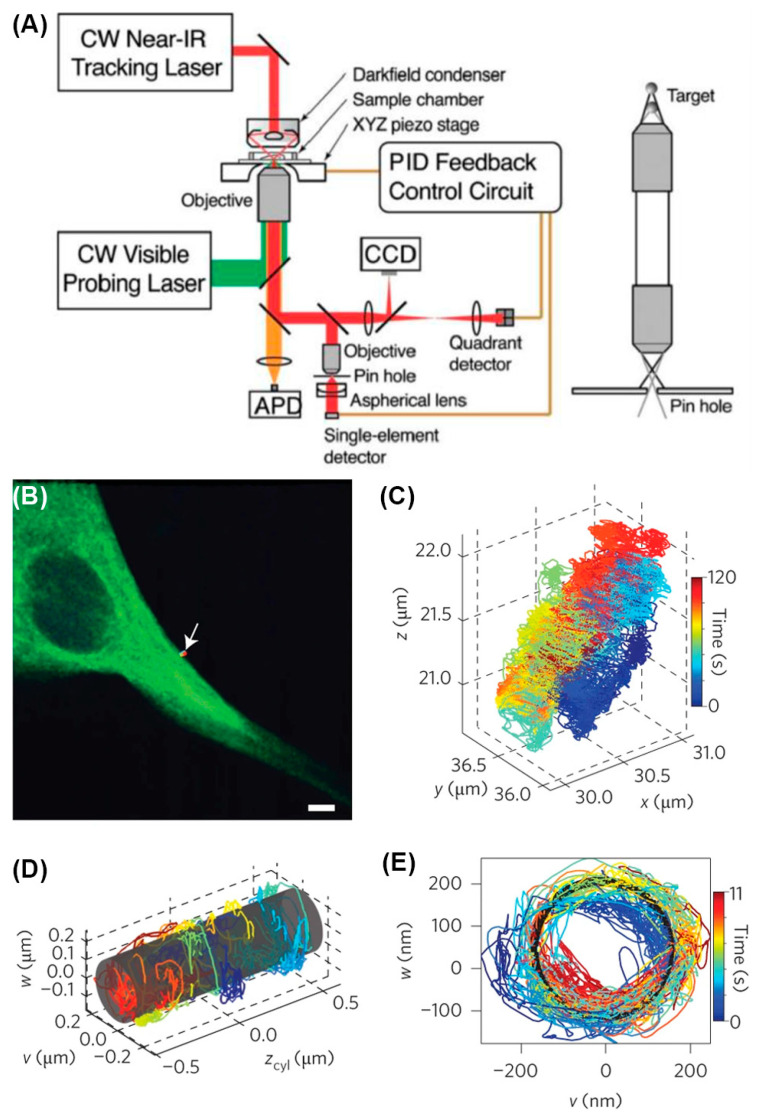
Multi-detector active feedback 3D tracking. (**A**) Schematic of the dark-field multi-detector active feedback tracking system developed by Cang et al. [120], which utilized a quadrant photodiode. Reprinted from Ref. [120] with permission of AIP Publishing. (**B**) Maximum intensity projected 2P-LSM data and high-resolution trajectory of a polystyrene–quantum dot–peptide nanoparticle (PS-QD-Tat particle) traveling along a filopodium at the cell edge. The white arrow indicates the location of the PS-QD-Tat particle. Scale bar, 5 µm. (**C**) High-resolution 3D trajectory from the image in (**A**) showing multiple cylindrical surfaces. (**D**) Cylindrical fitting of a short section of the trajectory in (**C**). (**E**) Cross-section of the cylindrical fit of (**D**), showing the cylinders that each have a diameter of ∼300 nm. Panels (**D**,**E**) share the same color scale bar. (**B**–**E**) Reprinted from Ref. [123] with permission of the authors. Copyright 2014, Springer Nature.

Yang and co-workers first developed multi-detector active feedback tracking based on a confocal microscope in 2006 [120]. In the initial implementation, the axial position is determined by the intensity level after a confocal pinhole, while the XY position is measured with a quadrant avalanche photodiode [120] (Figure 7A), updated to four SPC-APDs paired with two prism mirrors to split the signal in later iterations [121]. By implementing a nonpolarizing beamsplitter, a Wollaston prism, and a polarizing beamsplitter into this system, the orientational dynamics of gold nanoparticles can be measured [122]. In some interesting applications, individual Janus particle microscopic swimmers can be actively manipulated and navigated with an actuation laser. The active feedback system enables adaptive propulsion, enabling microscale particles to be navigated toward a target in three dimensions. Multi-detector active feedback tracking was also successfully applied to address biological questions, with simultaneous 3D contextualization using an additional two-photon laser scanning microscope (2P-LSM) that imaged the particle’s current focal plane [123] (Figure 7B–E). Although the frame rate of the 2P-LSM is relatively slow (~1 frame per second), the pixel dwell time is in the order of microseconds. At this timescale, both the particle and piezo stage are effectively stationary, resulting in no motion blur if images are constructed on a per-pixel basis. The pixels can be reassigned and overlaid with trajectories using the real-time stage position and relative pixel location. Integration of pixels along the entire trajectory yields 3D volumes of the cellular environment. In this work, polystyrene (PS) and semiconductor quantum dot (QD)-based fluorescent nanoparticles, modified with the Tat peptide derived from HIV-1, were used to act as a cellular delivery probe. By tracking exocellular virus-sized particles with this method, several phenomena have been observed and contextualized with cellular context, such as extracellular diffusion, diffusion on membrane protrusions, and particle binding on the cell membrane.

Another early example of modified detection active feedback tracking is a tetrahedral detection design by Werner and co-workers in 2007 [124]. In this method, two fiber-coupled SPC-APDs are placed at the image plane of a microscope such that the emission is split equally onto both detectors when the particle is centered along the horizontal direction. Similarly, two more fiber-coupled SPC-APDs are placed to detect vertical displacements. The two pairs of SPC-APDs are offset axially, and the difference between the paired signals is used to calculate the Z position. A high-speed piezoelectric stage is driven accordingly to complete the feedback loop. This method has been applied to track quantum dots or fluorescent nanospheres in a high background environment in 80% glycerol and in live cells [125].

One of the major drawbacks of modified detection methods is that the limited emission signal is split for detection. Given this split signal, brighter particles are usually required for a sufficient tracking signal. Despite this seeming limitation, the first active feedback single-fluorophore tracking was accomplished with the tetrahedral detection method. Tracking of individual organic dyes and FPs was demonstrated, with a diffusion coefficient of up to 1 μm^2^/s and a mean tracking duration of ~0.3 s [126].

The short trajectory duration, which is far less than the expected photobleaching time, indicates an undesired tracking performance when this method is applied to dim objects [125,126]. Due to the limited brightness of a viral particle labeled with an organic dye or fluorescent proteins, researchers used virus-sized, QD-labeled, Tat-coated fluorescent nanoparticles as a viral mimic to study viral infection, which inevitably sacrificed biological relevance compared to a pseudotyped lentivirus or a complete virion [123]. To date, there has been no application of modified-detection active feedback tracking to highly scattering or high-background environments, such as tissue. This may be because position estimation, which relies on balancing the signal across multiple detectors, would be significantly affected by high-scatter signals and uneven backgrounds. Therefore, modified detection methods may not be a suitable candidate for SVT in complex environments with high background.

### 3.2. Orbital Tracking

**Figure 8:**
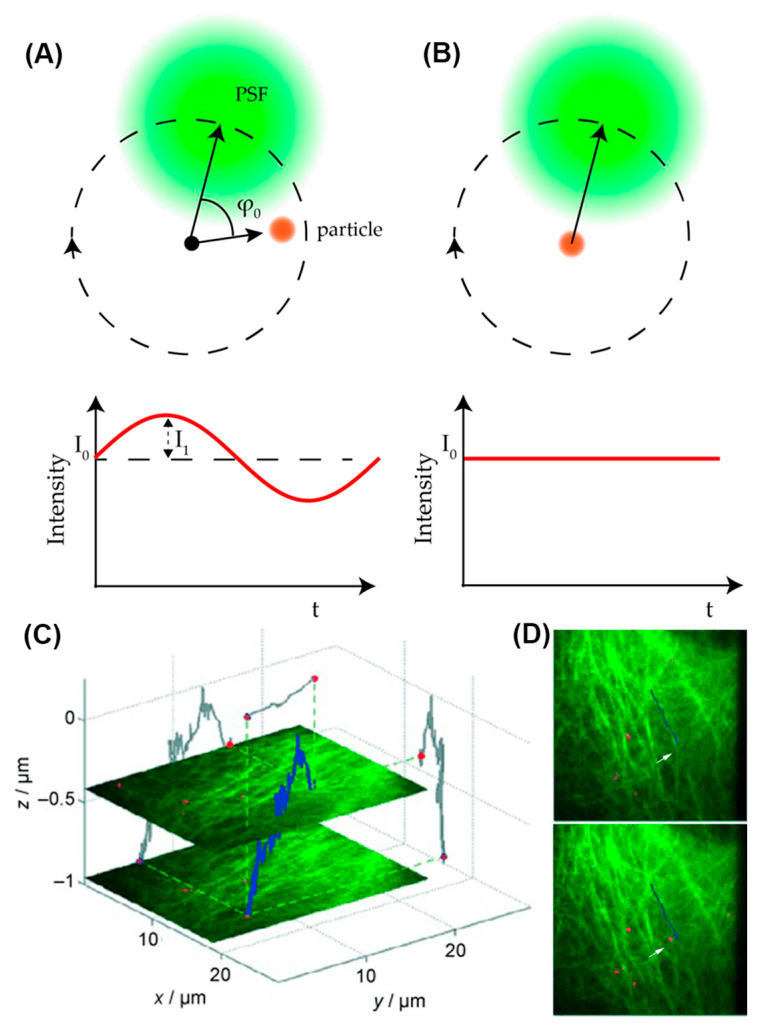
(**A**,**B**) Concept of orbital tracking. (**A**) Optical setup and 3D stereograms of two defocused 2*π*-DH-PSF combinations. (**B**) Three-dimensional trajectory of fluorescent microspheres in HeLa cells. (**A**,**B**) Reprinted from Ref. [56] under the terms of CC BY 4.0. (**C**,**D**) Orbital tracking of artificial virus in HUH7 cells contextualized with simultaneous wide-field imaging. (**C**) A 3D trajectory (blue) of an artificial virus (red) in a live HUH7 cell transfected with eGFP-tagged tubulin (green) along with two wide-field images taken at different z-positions during the measurement. 2D projections of the 3D trajectory are shown in gray on the respective axes. (**D**) Two snapshots from the movie showing (top) the virus travelling upwards along a microtubule fiber and (bottom) the virus switching to a different microtubule. Reproduced with permission from Ref. [131]. Copyright 2009, Wiley.

Instead of using multiple detectors, other active feedback tracking methods utilize patterned excitation. In patterned excitation methods, the laser excitation is carefully engineered temporally such that the time arrival of collected photons can be used to infer the real-time position of the particle. Orbital tracking was the pioneering approach among patterned excitation methods [127]. In most implementations, a circularly rotating laser beam in the XY plane is generated with two galvanic mirrors. The scan diameter is typically chosen to be close to the PSF size. When the particle is located in the center of the scan, its intensity stays constant (Figure 8A). When the particle moves towards the edge of the scan, the detected intensity will fluctuate periodically with an amplitude directly proportional to the particle center distance in the XY plane (Figure 8B), which can be extracted with a lock-in amplifier or fast Fourier transformation [128]. In 3D orbital tracking methods, the scan frequency, typically ranging from tens to hundreds Hz, defines the temporal resolution.

Tracking in the third dimension has been achieved using a piezoelectric objective lens positioner, which is moved to detect two different focal planes, one above and one below the particle [129]. This approach has been improved by replacing the stage with an electrically tunable lens (ETL), resulting in significantly improved temporal resolution to 8 ms with a demonstrated axial tracking range of up to 300 μm while tracking fluorescently labeled loci in live cells [130]. The Lamb group adopted a hybrid approach in which the lateral particle position is determined by the orbiting laser position, while the axial position is determined by separate detectors, one focused above and one below the focal plane. This modification eliminated the need for an axial scan, the slow step in the process, resulting in improved localization precision and temporal resolution [131]. Using this approach, along with a simultaneous wide-field imaging channel, the Lamb group visualized the transport of fluorescent-labeled artificial viruses, DNA polyplexes in this case, in HUH7 cells (Figure 8C,D). More recently, this approach was advanced to be able to track two independently diffusing particles simultaneously by driving the piezoelectric stage jump between two particles continuously [132]. This is the first such active feedback method to measure two particles simultaneously and an important advance for future active feedback SVT methods.

Despite all the successful modifications and applications of orbital tracking, this method is still fundamentally limited by the scan frequency and the scanning size limited to the PSF. The scan frequency and size together determined the capability of tracking fast-diffusing particles. Also, orbital tracking cannot function properly in the presence of a high and uneven background, which can easily distort the intensity fluctuation and lead to tracking failure [133,134].

### 3.3. Tetrahedral Excitation Tracking

Similar to the tetrahedral detection tracking methods mentioned above, tetrahedral excitation tracking methods use four alternating laser pulses for fluorophore excitation. As the name suggests, the four laser foci are arranged in a tetrahedral pattern, with only one spot active at any given time. Emitted photons are then detected by a single SPC-APD. The time arrival of the photons indicates which laser spot was active at the time of excitation. Active feedback is then employed to recenter the particle by balancing the photon arrivals from each spot.

**Figure 9:**
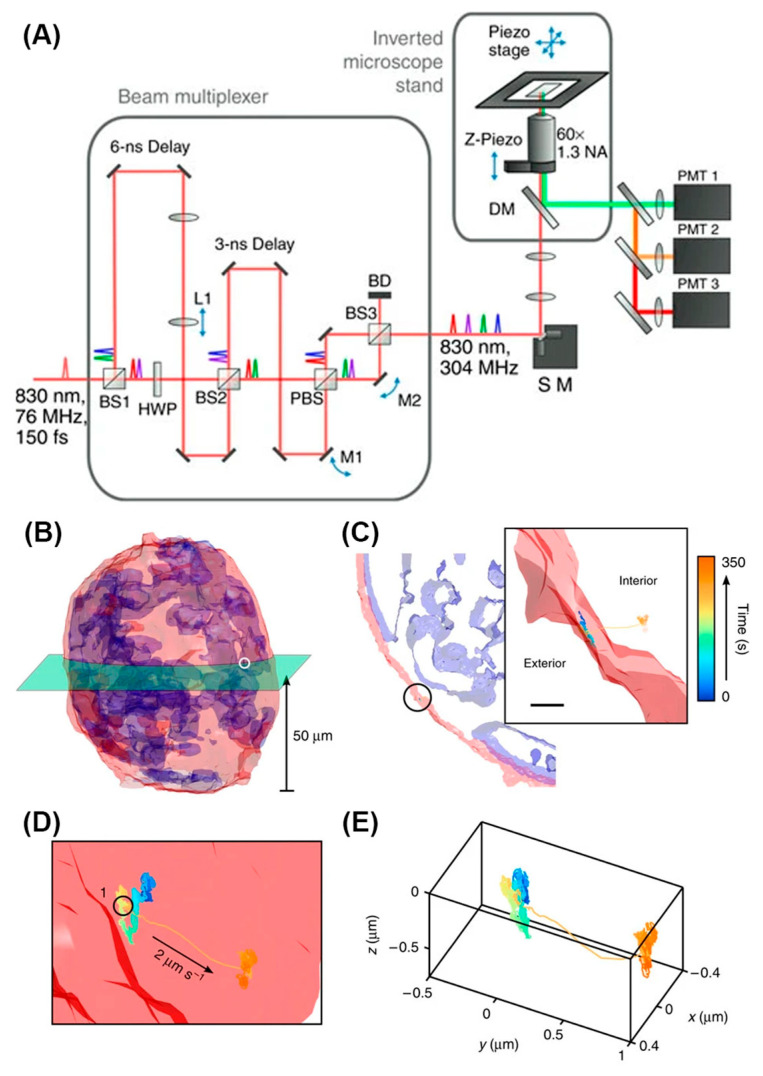
(**A**) Schematic of the TSUNAMI method. Spatiotemporal multiplexing is enabled through an optical system that utilizes two beam splitters (BS1 and BS2) to generate four beams, which can be quasi-independently controlled via mirrors (M1 and M2). Physical delay lines provide temporal separation. In this case, 6.6 ns (2 m) and 3.3 ns (1 m) path length delay lines create four beams with a period of 3.3 ns corresponding to an even division of the fundamental 13 ns period generated by the laser source. Tracking actuation is performed using scanning mirrors (SM) and an objective focusing stage (z-piezo). (**B**–**E**) Application of deep 3D-SPT in a spheroid model. (**B**) 3D isocontour of tumor spheroid taken with 2P laser scanning microscopy staining for the plasma membrane (red) and nuclei (blue). The highlighted slice (at 50 μm depth) shows the plane of the EGFR internalization trajectory. (**C**) Isocontour model of the green slice in (**B**). The inset shows a zoomed-in view highlighting the spheroid boundary and the placement of the trajectory as it transports into the spheroid. (**D**) Zoom in of (**C**). (**E**) Isolated particle trajectory. Reprinted from Ref. [36] under the terms of CC BY 4.0.

The first tetrahedral excitation tracking was achieved using four pulsed laser diodes [135]. Four laser beams were merged with beamsplitters and overlapped in a tetrahedral geometry at the focus of the objective lens. As an improvement of this method, the tracking of single particles using the nonlinear and multiplexed illumination (TSUNAMI) method used one mode-locked Ti:Al_2_O_3_ pulsed laser to replace four laser diodes and achieve two-photon excitation for better performance in thick biological samples [36] (Figure 9A). The laser pulses have a 3.3 ns delay between each other and similarly form a tetrahedral excitation volume after passing through the objective lens. In TSUNAMI, the environmental context was captured with asynchronized two-photon 3D images. Thanks to the depth provided by two-photon excitation for both tracking and imaging, this method has been used to visualize epidermal growth factor receptor (EGFR) internalization pathways in A431 tumor spheroids with a diameter of 100 μm (Figure 9B–E). This method is also capable of tracking freely diffusing particles with diffusion coefficients of 2–4 μm^2^/s with a tracking duration of 1–2 s.

### 3.4. MINFLUX Single-Molecule Tracking

The advances of super-resolution microscopy have pushed the precision of microscopy down to the nanometer level and beyond. The most well-known Nobel Prize-winning methods, STED, PALM, and STORM, shared a similar philosophy of exciting one fluorophore while its neighbors remained unexcited. However, being able to localize the excited fluorophore is equally important. Developed by the Hell group, the concept of minimal emission fluxes (MINFLUX) nanoscopy was developed to acquire precise localization of fluorophores with a minimum number of photons [136]. Unlike other localization methods that favor a stronger signal, MINFLUX utilizes a donut-shaped beam with a minimum intensity for localization and targets at the intensity minima. This technique has been demonstrated in live cells [137]. Further, it has been demonstrated that the scan size can be shrunk down to provide precision down to the molecular level [138]. MINFLUX has been demonstrating its strength in single-molecule tracking in biological contexts and has recently been applied to directly visualize motor protein stepping in live cells [139,140]. A recent application has achieved imaging up to 80 μm deep in a fixed tissue while retaining sub-ten-nanometer localization precision [141].

MINFLUX has also shown great synergy with other microscopic methods, including pulsed excitation, time-resolved detection, single-molecule FRET (smFRET), and 4Pi configuration. Masullo et al. achieved a novel pulsed interleaved MINFLUX (pMINFLUX) by modifying the excitation path of confocal microscopes with a pulsed laser plus a time-correlated single-photon counting (TCSPS) detection [142]. In this setup, fast-scanning optics or an FPGA are not required, while lifetime information can be additionally extracted. Nanometer precision was achieved in 3D by combining pMINFLUX with graphene energy transfer and DNA-PAINT [143]. pMINFLUX can further combine with smFRET and lifetime multiplexing to enable simultaneous nanometer-precise tracking of multiple fluorophores, bridging the resolution gap between smFRET and single-molecule tracking [144]. Recently, 4Pi MINFLUX, which integrates MINFLUX localization with two opposing objective lenses, has maximized spatiotemporal precision [145].

As MINFLUX was designed for imaging and localizing single-molecular-sized objects, its scanning pattern was intended to be small (typically ranging from 50 nm to 300 nm) to extract the best precision. However, the narrow scanning range limits the tracking speed to about 1 μm^2^/s since fast-moving particles can easily move out of the tracking region. Particularly, the axial scanning size is often as narrow as the lateral scan for 3D-MINFLUX, which limits the working z-range and creates fundamental barriers for its application in tissue [137,138]. Another major limitation of MINFLUX is its relatively weak resistance to the undesired signal–background ratio, which could distort the localization algorithm and affect its tracking performance.

### 3.5. 3D Single-Molecule Active Real-Time Tracking

**Figure 10:**
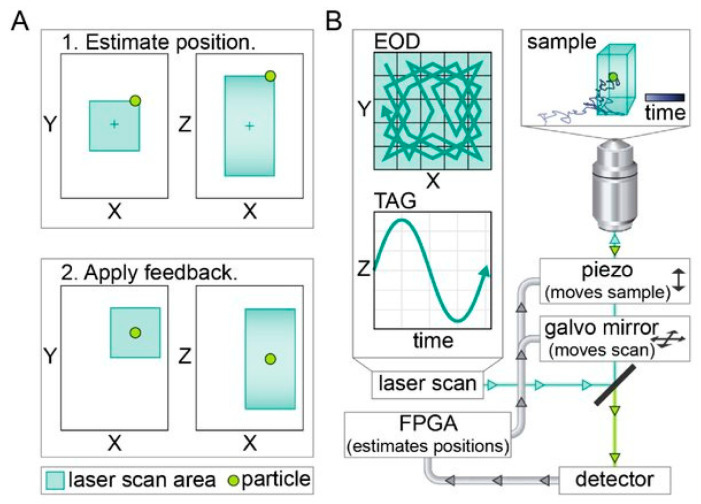
(**A**) General process of 3D active feedback particle tracking. First, the particle position is estimated. The position estimate is then used to recenter the tracked particle within the tracking observation volume. (**B**) Simplified diagram of experimental implementation. An electro-optical deflector (EOD) generates a 2D knight’s tour. A tunable acoustic gradient (TAG) lens scans the laser focus along Z. These form the tracking observation volume, a 1 × 1 × 2 pm box. Position estimates are calculated using a field programmable gate array (FPGA). Then, feedback is applied using a piezo objective nanopositioner along Z and a galvo mirror to deflect the entire scan pattern along X and Y. Reprinted from Ref. [161] with the permission of the authors. Copyright 2023, Society of Photo-Optical Instrumentation Engineers (SPIE).

An active feedback method that has been applied to SVT is 3D Single-Molecule Active Real-Time Tracking (3D-SMART), developed by Hou et al. [146,147]. In this method, the laser is deflected in three dimensions with a pair of electro-optic deflectors (EODs) and a tunable acoustic gradient (TAG) lens (Figure 10B) [148]. The lateral scanning pattern is a 1 μm × 1 μm knight’s tour pattern generated by the EOD pair, while axial scanning is achieved by the TAG lens over a range of ~2 µm. The scanning area is noticeably larger than the methods listed above, making 3D-SMART particularly well-suited for tracking rapidly diffusing particles, maintaining trajectories through emitter dark periods, and compensating for the lag of the repositioning device. The rapid 3D scanning rate (50 kHz lateral, 70 kHz axial) enables high temporal resolution. Fluorescently labeled particles or even single molecules are excited by the rapid 3D scanning laser, and the photons are collected by a single-photon counting avalanche photodiode (APD), which is used to estimate the real-time position of the particle with an assumed Gaussian density Kalman filter [149]. An integral feedback controller is applied to the piezoelectric nanopositioner or galvo mirror to lock the particle in the center of the scan volume (Figure 10A). 3D-SMART is capable of tracking a variety of highly diffusive particles (up to 10 μm^2^/s) for minutes in duration, and this method has been applied to study viral first contact [150], lipid nanoparticle diffusion and intracellular trafficking [151,152], in situ characterization of the nanoparticle protein corona [153], living polymerization [154], hopping diffusion in porous networks [155], nanoscale force mapping by tracking sliver nanoparticles in live cells [156], and single-particle manipulation and electrophoretic mobility measurement by applying an electric field [157]. A recent extension of 3D-SMART incorporated fluorescence spectral readout, enabling simultaneous single-molecule 3D tracking and monitoring of local physicochemical dynamics [158,159]. Similar to MINFLUX, 3D-SMART and other active feedback tracking methods with patterned excitation can also benefit from modifications to the scanning pattern. Zhang et al. demonstrated that off-center sampling could utilize photons more efficiently while yielding better precision and accuracy [160].

### 3.6. 3D Tracking and Imaging Microscopy

Active feedback SVT methods, such as 3D-SMART discussed above, lack simultaneous contextualization of the cellular environment during tracking. Without simultaneous wide-field volumetric imaging, measured viral dynamics will lack important cellular context. However, implementing simultaneous contextualization is difficult with active feedback methods. Unlike image-based methods, the sample in active feedback methods is usually moving to track the particle in real time, meaning camera-based imaging would suffer from motion blur. Also, with the piezoelectric stage occupied for tracking, it is not possible to use it for z-stack and axial 3D imaging, as is common in LSCM and SPCM. To enable tracking of fast viral motions in context, 3D-SMART has been integrated with synchronized contextual imaging via 3D Tracking and Imaging Microscopy (3D-TrIm). 3D-TrIm was recently developed by Johnson et al. to track the extracellular dynamics of viral particles with high temporal resolution while simultaneously capturing the surrounding 3D cellular context [21]. In this work, vesicular stomatitis virus (VSV)-G-pseudotyped lentiviruses were chosen for their broad cellular tropism, owing to the ubiquity of the low-density lipoprotein receptor (LDLR). The lentiviruses were internally packaged with fluorescently labeled HIV-1 viral protein R (Vpr) to minimize unwanted inhibition induced by external labeling [162].

In 3D-TrIm, while fluorescently labeled viral particles are tracked by 3D-SMART, a two-photon volumetric imaging system called 3D Fast Acquisition Scan by z-Translating Raster (3D-FASTR) acquires images of the cellular environment [163]. 3D-FASTR is based on 2P-LSM, which means individual pixels are synchronized with the real-time particle location as discussed above [123]. However, using 2P-LSM alone restricts observation to just the focal plane currently containing the tracked particle. To achieve volumetric imaging, 3D-FASTR uses an electrically tunable lens (ETL) to rapidly scan the focus axially to generate a raster scan pattern in Z. This creates a rapidly but incompletely sampled 3D volume, where unsampled voxels are interpolated from their sampled neighbors. The result is a volumetric image of the environment surrounding the tracked particle. With synchronization between tracking and imaging, viral dynamics can be contextualized with fluorescently labeled extracellular and intracellular environments. As a demonstration of 3D-TrIm’s ability to perform contextualized 3D-SVT, diffusivity of virus-like particles (VLPs) was quantified as a function of distance from the cell surface, and millisecond-scale transient contacts were observed and quantified.

**Figure 11:**
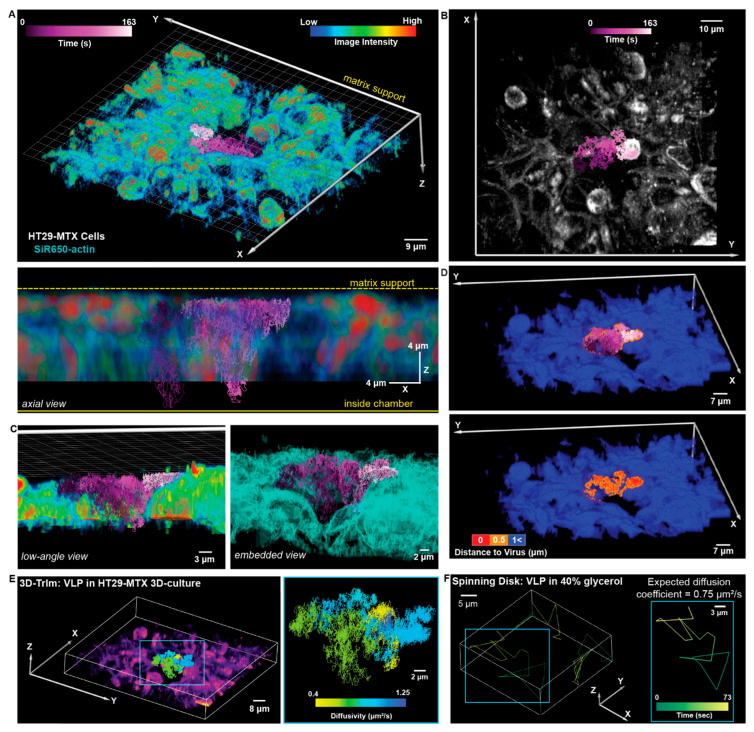
(**A**) Top, 3D reconstruction from a 4D dataset covering 10 local volumes, at 10 frames per volume, of suspended HT29-MTX cells grown on inverted matrix support and labeled with SYTO61. Coregistered VSV-G VLP trajectory color-coded by time. Bottom, lateral (xz) view. (**B**) Top-down (xy) maximum intensity projection with trajectory overlay. (**C**) Right, magnified cut-through view showing VSV-G VLP confined within vacancy as it diffuses around the edge of tightly packed epithelial cells. Left, isosurface render of cells highlighting sample density. (**D**) Top, the distance of the trajectory to the cell surface is projected as a distance map on the cell volume, with the trajectory color-coded by time overlayed. Bottom, volume with trajectory omitted, showing areas of close contact on the cell surface. (**E**) Left, the resulting trajectory is color-coded by a diffusion coefficient, using mean squared displacement and change-point analysis. Right, trajectory with volume omitted, showing a highly sampled trajectory across a large range in diffusivity. (**F**) The same fluorescently labeled VLPs (eGFP-Vpr VSV-G) were tracked in 40% glycerol (*v*/*v*) on an Andor Dragonfly Spinning Disk Confocal microscope, with a camera exposure time of 40 ms. A single volume (~8 μm split into 16 z planes) was sampled continuously. The resulting trajectories over the entire acquisition period are shown and color-coded by time. Reprinted from Ref. [21] with permission of the authors. Copyright 2022, Springer Nature.

Beyond monolayer cell models, 3D-TrIm was applied to tissue models comprising HT29-MTX cells on a semipermeable membrane support (Figure 11). HT29-MTX is a type of mucous-secreting intestinal epithelial cell model that also forms tight intracellular junctions and is widely used in studying cell–pathogen interactions [164]. It was uncovered that the VLPs underwent confined diffusion but could still travel more than 10 μm along the *Z*-axis. Also, due to the msec temporal resolution of the viral trajectories, 3D-TrIm revealed significant changes in viral dynamics while diffusing through these 3D epithelial models. The high spatiotemporal resolution of 3D-TrIm offers a tool for studying the virus–epithelium interaction at a level of detail possible with conventional image-based methods.

## 4. Novel Virus Labeling Methods Facilitate Single-Virus Tracking

Since viruses are not natively fluorescent, proper fluorescent labels of viral components are needed for SVT. To achieve long-term tracking without affecting virus infectivity, an ideal fluorescent label should possess excellent brightness, photostability, and biocompatibility. Fluorescent probes that meet these criteria provide high spatiotemporal precision trajectories over the complete infection cycle.

Many categories of fluorescent labels are currently used for virus labeling, including organic dyes, fluorescent proteins (FPs), and fluorescent nanoparticles (NPs), such as quantum dots (QDs). The photophysical characteristics and the physical size of each type of probe lead to specific pros and cons of each labeling approach. Generally, organic dyes and FPs possess great brightness and small probe size but suffer from poor photostability [165]. Additionally, FPs are introduced to viruses by genetic engineering, which limits their application to tracking non-protein components or virions collected from infected tissue. Fluorescent NPs, especially QDs, have been widely used for SVT for their outstanding quantum yield and resistance to photobleaching [166]. Their larger size than other probes (up to 20 nm) [166], however, should be considered, since NPs can easily create steric hindrance and interrupt native cell–virus interactions. The size effect of quantum dots on SVT is further discussed in Section 4.3.

The viral infection cycle can span hours [167,168,169], including but not limited to extracellular diffusion, binding, internalization, uncoating, nuclear import, and intranuclear diffusion. Therefore, an appropriate label for long-term SVT should possess a stable hour-long fluorescence emission. In this section, we will discuss the major approaches to extending the tracking duration for fluorescent proteins, organic dyes, and fluorescent nanoparticles.

### 4.1. Fluorescent Proteins

FPs are typically linked to specific viral components through genetic engineering. Viral target proteins include envelope, matrix, capsid, and ribonucleoprotein. Several FPs, such as EGFP, EYFP, and mCherry, have been incorporated into viral proteins for single-virus tracking [28,170,171,172,173,174]. Highly photostable and bright FPs are favorable for extending the virus tracking duration and continuously observing virion behavior. However, photostable FPs can barely be obtained without sacrificing brightness (e.g., variants of mOrange, TagRFP, and mScarlet) [175,176], except for several bright and photostable FPs (e.g., Azurite, EBFP2, and mGold) [177,178,179,180]. This trade-off arises from the participation of molecular oxygen in both chromophore maturation and decomposition. The high affinity of the FPs’ chromophores to oxygen contributes to their fast maturation and maximal brightness [165], but oxygen also reacts with the chromophores in their excited states and leads to fast photobleaching [166]. The development of bright and photostable FPs is essential for long-term SVT.

A recently published naturally dimeric green-emitting fluorescent protein, StayGold, has shown promise as an extremely photostable FP that may enable long-term SVT [165]. Photobleaching assays of both purified proteins and proteins expressed in live cells have revealed significantly greater photostability and brightness compared to other conventional FPs. Specifically, the photobleaching half-life of previously reported FPs is exclusively shorter than 700 s, while StayGold exhibits a half-time of over 10,000 s under the same excitation power. Further understanding of the underlying mechanisms of the interaction between molecular O_2_ and the StayGold chromophore may guide the development of more photostable FPs. Overall, this remarkable photostability makes StayGold a promising candidate for fluorescent SVT on infection-relevant timescales. In practice, fusing StayGold with viral protein R (Vpr) of VSV-G-pseudotyped lentiviruses has yielded enhanced photostability and significantly prolonged tracking duration of single viral particles in live cells, which enables the analysis of long-term viral motions, such as intracellular linear and spherical trafficking [181]. More recently, the combination of 3D-TrIm with highly photostable StayGold-labeled SARS-CoV-2 VLPs revealed a previously hidden, actin-dependent membrane trafficking mode during viral entry, showing that viruses undergo prolonged 3D surface exploration before internalization [182]. Further development of monomeric StayGold potentially eliminates the unintended dimerization of targeted molecules while maintaining brightness and photostability [183,184,185]. More recently, several other FPs, such as yellow fluorescent protein mGolds and mGoldt and red fluorescent protein mScarlet3-H, have also demonstrated promising photostability and expanded the spectral availability [186,187].

### 4.2. Organic Dyes

**Figure 12:**
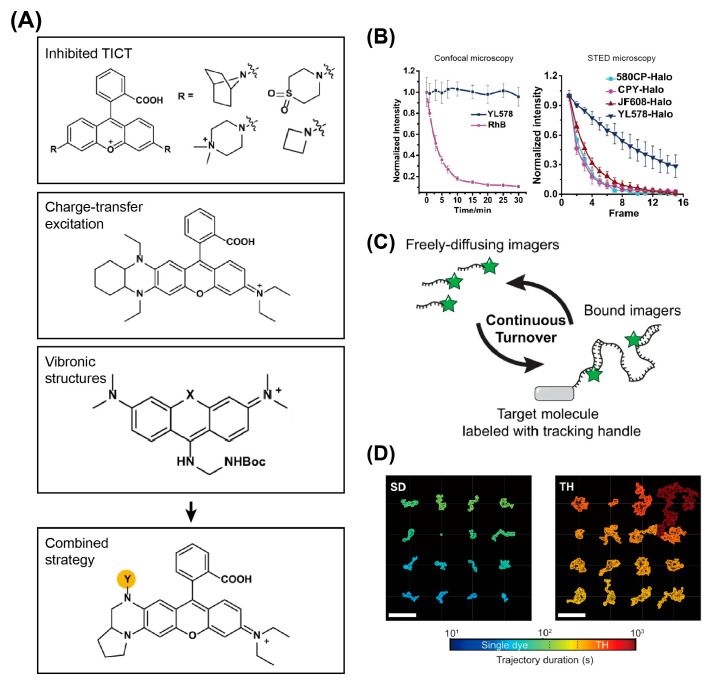
Strategies for extending the tracking duration of organic dye-labeled virus. (**A**) Combining TICT inhibition and vibronic structure for designing highly photostable organic dyes. (**B**) Comparison of the photostability of engineered organic dye YL578 with other commonly used dyes with live-cell confocal or STED microscopy imaging. (**A**,**B**) Adapted from Ref. [192] under the terms of CC BY 4.0. (**C**) Schematic illustration of DNA-PAINT for hour-long single-particle tracking. (**D**) Representative trajectories of single-dye (SD) or DNA-PAINT tracking-handle (TD) probes on a supported lipid bilayer. Trajectories were color-coded by tracking duration. Scale bar: 10 µm. (**C**,**D**) Adapted from Ref. [195] under the terms of CC BY 4.0.

Organic dyes are also commonly used for virus labeling and tracking due to their small size, biocompatibility, and straightforward labeling procedures. Cyanine dyes (e.g., Cy3 and Cy5) and Alexa Fluor have been widely used for covalent labeling of viruses [188,189,190]. Lipophilic dyes (e.g., DiD and DiO) are able to insert into and uniformly label viral envelopes through hydrophobic interactions [25,191]. However, the observation time of a particle labeled with a single dye is restricted to a few seconds before photobleaching [30], so large numbers of dyes are needed per virion. Recent efforts have focused on rationally engineering organic dyes for increased photostability. Twisted internal charge transfer (TICT) is a type of intramolecular charge transfer (ICT), in which internal bond rotation leads to energy relaxation of the excited state without photon emission. This non-radiative decay reduces the brightness as well as the photostability of dyes through radical production with molecular oxygen. To inhibit TICT, Jiang et al. modified rhodamine B (RhB) by introducing a pyrazine moiety and electron-withdrawing groups, both of which increase the energy barrier of TICT by limiting C-N bond rotation and reducing electron density in the fluorophore scaffold [192] (Figure 12A,B). YL578, the engineered derivative from RhB, exhibited negligible photobleaching within 30 min in live-cell imaging, while the photobleaching half-time of RhB was only 5 min. Additionally, Kim et al. developed a photostable PF555 dye from photoblued TSCy5 [193]. PF555 showed a 10- to 50-fold increase in photobleaching half-time compared to other common organic dyes with similar emission spectra, which was attributed to its spontaneous recovery to the bright state from the dark state. PF555 was then specifically tagged to live-cell epidermal growth factor receptor (EGFR) for hour-long single-particle tracking.

Modifying the assembly and packaging of organic dyes also facilitates long-term tracking by increasing their brightness or photostability. Liu et al. labeled the Japanese encephalitis virus (JEV) genome for SVT with multiple Cy5- and BHQ-modified molecular beacons (MBs) [31]. The MBs are short nucleic acid strands with stem-loop structures that maintain closely spaced fluorophores and quenchers. The fluorescence of MBs is suppressed by the quencher until they hybridize with their target, opening the stem and restoring fluorescence. Introduction of multiple MBs that bind to different viral genome sites could enable a high signal-to-noise ratio and dramatically extend photobleaching time for SVT. Instead of applying multiple single-dye probes, Niekamp et al. developed a DNA-based “FluoroCube” with multiple dyes in a single probe [194]. The programmable DNA-assisted Cy3 dye assembly yields an over 54-fold increase in *t*_1/2_ compared with single Cy3-modified double-strand DNA, possibly through a FRET mechanism. The assembly of several other dyes into DNA FluoroCubes also produces a highly photostable probe with a longest observed half-life of 1834 s. These FluoroCubes can be easily attached to proteins of interest through functional groups such as HALO-ligand, benzyl guanine (BG) for SNAP-tag, thiol, biotin, or amine groups. Collectively, considering the brightness, photostability, and the small size (6 nm) of DNA fluorocubes, these results highlight the possibility of incorporating this probe into viral proteins for long-term SVT.

Unlike extending the lifetime of a single dye, continuously replacing dyes that have cycled into a dark state with fresh ones could be an alternative strategy to mitigate photobleaching-induced signal loss. Stehr et al. applied and re-purposed the idea of DNA-PAINT (Points Accumulation for Imaging in Nanoscale Topography) for hour-long single-particle tracking [195] (Figure 12C,D). This DNA-mediated probe exchange enables the fast replacement of fluorophores on the target molecule before the photobleaching so that a particle could be observed and tracked with a negligible fluorescence signal change for 30 min. Overall, the combination of these techniques discussed in Section 4.2 demonstrates the possibility of tracking a single virus for hours with minimal interference to its natural behavior.

### 4.3. Fluorescent Nanoparticles

Fluorescent semiconductor quantum dots (QDs) have been used to image and track viruses owing to their superior photostability, brightness, narrow emission spectra, and large Stokes shifts [166]. QDs’ excellent brightness provides a high signal-to-noise localization for viral trajectories, while the large Stokes shift helps ensure high signal-to-background. Their strong resistance to photobleaching enables the observation of a single virus for several hours, shedding light on the complete timeline of viral infection. Furthermore, due to their wide excitation range and the narrow emission spectrum (typically 20 to 30 nm of full width at half maximum) [196], QDs with different sizes can be excited with a single wavelength of light and generate many well-resolved emission colors. Therefore, multiplexed imaging of several viral component QDs with different emission wavelengths is theoretically possible.

**Figure 13:**
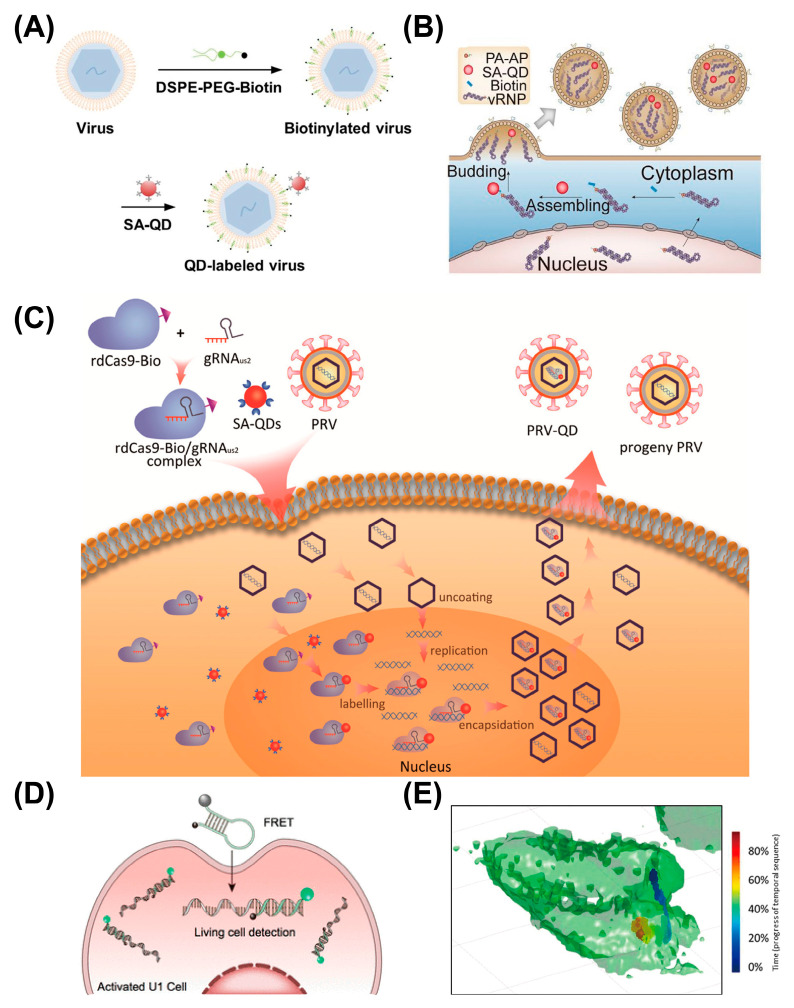
Representative strategies for labeling viruses with fluorescent nanoparticles. (**A**) Schematic illustration of viral lipid membrane labeling with QDs. DSPE group guides hydrophobicity-based lipid recognition. Reprinted from Ref. [197] under the terms of CC BY 4.0. (**B**) Schematic illustration of vRNP-specific QD labeling with BAP fusion. Reprinted from Ref. [167] under the terms of CC BY 4.0. (**C**) Schematic illustration of viral genome labeling with the CRISPR-dCas9 system. Reprinted with permission from Ref. [209]. Copyright 2020, American Chemical Society. (**D**) Schematic illustration of labeling the viral genome with nanobeacons modified by QDs (green) and quenchers (black). Reprinted with permission from Ref. [210]. Copyright 2019, American Chemical Society. (**E**) The trajectory of giant QD-labeled HIV-1 VLP and cell images describing the viral extracellular diffusion stage by 3D-MRM. Reprinted from Ref. [221] under the terms of CC-BY-NC-ND.

Biotin–streptavidin (SA) interaction is a powerful tool to specifically conjugate SA-functionalized QDs to virus particles. Viral membranes can be biotinylated by introducing lipophilic tail-modified biotin, and SA-QDs can specifically bind the viral envelope for single-virus tracking [197] (Figure 13A). However, given the larger size of QDs relative to FPs and small-molecule dyes, QDs labeled on the viral surface might hinder the natural behavior of viruses, including their interactions with receptors and intracellular protein trafficking. QD labeling of proteins has been shown to affect their diffusive behavior [198,199,200]. Although evidence shows that surface QD labeling has little effect on virus infectivity [201,202], further study is needed on the difference in virus behavior, such as their diffusion and binding events, compared to the non-labeled viruses. One solution to this potential problem is decreasing the size of QDs via surface engineering [203] or synthesizing QDs with different materials [204,205]. However, with viral surface labeling, membrane fusion after the virus entry will lead to the dissociation of QDs [206], so envelope labeling is restricted to early parts of the infection cycle.

Intraviral QD packaging has been proposed as an alternative strategy to minimize perturbation of the virus–cell interaction. Qin et al. encapsulated QD-conjugated viral ribonucleoprotein complexes (vRNPs) within influenza A viruses (IAV) for real-time tracking of the uncoating process [167] (Figure 13B). Here, the biotin acceptor peptide is fused to the PA subunit of the IAV vRNP to bind biotin. After biotin insertion, the SA-QDs are delivered into cells using liposomes and specifically label vRNPs. The multicolor labeling on IAV’s envelopes and different vRNP segments revealed the mechanisms of IAV uncoating and vRNP trafficking. The fusion of other peptide tags (e.g., His tag and LplA tag) into viral proteins, followed by the conjugation between these tags and modified QDs, also allows the site-specific QD labeling of viruses [207,208].

Oligonucleotides can also enable viral DNA or RNA labeling and tracking. The complex of nuclease-deactivated CRISPR-Cas9 (dCas9) and guide RNA (gRNA) retains the ability of recognizing target DNA without cleaving it. Thus, dCas9 provides a great platform for gRNA-guided virus labeling. Biotinylated dCas9 protein with gRNA has been applied for specifically conjugating SA-QDs to the US2 gene of pseudorabies virus [209] (Figure 13C). This CRISPR-based labeling enables the single-virus tracking of pseudorabies virus entry and infection mechanisms.

Like the molecular beacons (MBs) discussed in Section 4.2, QD-modified MBs can label viral DNA or RNA by the same principle. MBs modified by a QD and a BHQ quenching group are only activated and fluorescent after their binding with a target nucleic acid, and thus can be used for tracking a single viral particle [210] (Figure 13D). Modifying MBs with upconverting nanoparticles (UCNPs) provides another approach to continuously observing virus particles. The UCNPs reported by Pang et al. possess excellent resistance to photobleaching and can be visualized with 550 nm emission under near-infrared (NIR) excitation (980 nm, 500 mW/cm^2^) for 12 h [211]. Due to the reduced scattering efficiency and larger penetration depth compared to visible light, the NIR excitation pattern of UCNPs enables the long-time and deep-tissue IAV tracking inside the mouse lung tissue. Recently, UCNPs were also utilized for live-cell SPT of EGFRs and glycan components [212,213].

Despite the great optical properties of QDs, their application to single-virus tracking still suffers from their intrinsic fluorescence intermittency (FI or blinking) [214]. FI interrupts the photon emission from these fluorescent NPs by transitioning the system into a long-lived dark state with a low emission rate [215]. Although FI is reversible, this fluorescent blinking could influence the fluorescence intensity of viral particles and the interpretation of SVT trajectories. Several strategies have been reported to suppress the blinking of fluorescent NPs [216,217,218,219]. Nonblinking giant QDsCdSe/CdS core–shell quantum dots [220] have been used for labeling and tracking single HIV-1 VLPs with an active feedback tracking microscope called 3D multi-resolution microscopy (3D-MRM) [221] (Figure 13E), which enables long-term single-virus tracking with high temporal resolution.

## 5. Outlook

The past few decades have witnessed the great value and potential of SVT in uncovering the details of the viral infection process. With the flourishing development of optical techniques, including new hardware and methodologies, there has been a huge leap in the spatiotemporal resolution, penetration depth, and resistance to high background. In this review, we have elaborated on the recent advances in single-particle tracking towards the goal of investigating the viral infection process in the epithelium, including the development of image-based methods, active feedback methods, and novel virus labeling techniques.

Among the methods discussed, we would like to highlight some recent advances towards the realization of tissue-level SVT. 3D-TrIm microscopy has demonstrated its potential for visualizing both intracellular and extracellular viral diffusion with fast tracking and good tolerance to depth and complex backgrounds [21]. MINFLUX nanoscopy is state-of-the-art in the field of single-molecule tracking, which conceptually avoids some obstacles of fluorescent microscopy, such as photobleaching and wavelength [136]. Though whether MINFLUX can be transplanted into SVT in tissue environments remains unclear, and its slow tracking speed restricts its capability of tracking free particles, its unprecedented spatiotemporal resolution has already been a game changer in single-molecule tracking [139,140]. Light-sheet microscopy may not be the most suitable method for SVT, but it offers advantages for tissue imaging, as mentioned in Section 2.5. There have been some more recent advances in tissue imaging that may eventually prove to be suitable candidates for SVT, including multiview confocal super-resolution microscopy from Shroff and co-workers [222] and light bead microscopy from Vaziri and co-workers [223]. In parallel, methodological innovations, such as 4Pi self-interference SPT [224], surface plasmon resonance microscopy-based label-free SVT [225], single-molecule super-resolution imaging using a reversed virus–cell configuration [226], and deep learning-assisted analysis of SVT trajectories [227,228], have further expanded the technical repertoire, offering enhanced precision and automation of viral entry dynamics.

Despite all the advances in SVT, there is no well-rounded method for studying viral–epithelial interactions. As discussed in Section 1.3, the thickness of 3D epithelial models is one of the major obstacles in trans-epithelial SVT. Most existing microscopic methods fail to well visualize viral diffusion in this complex environment without further modification. The major obstacles are the limited penetration depth of visible light through thick tissue and the degradation of the PSF due to scattering and spherical aberration.

Multiphoton microscopy is a widely used technique to address these challenges and image through considerably thick biological samples in vitro and in vivo. In multiphoton microscopy, multiple photons are absorbed simultaneously for excitation, and this multiphoton absorbance can only be achieved at the focal point. This eliminates the out-of-focus emission, which allows the collection of high signal-to-background images through highly scattering thick samples. As a further benefit, the narrow excitation region restricts photobleaching of out-of-focus objects within the sample. Xu and co-workers demonstrated successful two-photon in vivo imaging to a depth of 1.6 mm in 2011 [229]. Multiphoton microscopy can also easily be implemented into active feedback tracking microscopy. TSUNAMI, mentioned above, took advantage of two-photon excitation and tracked particles at a depth of ~100 μm in tumor spheroids [36].

While multiphoton microscopy facilitates light penetration through thick samples, the emission signal still suffers from absorption and scattering within the thick sample. Adaptive optics, which corrects for optical aberrations, is a strong tool to restore imaging quality after the aberrations from cells and tissues [230]. Briefly, adaptive optics has two key components: sensors (sometimes in the form of the image itself), which measure aberrations, and correctors, which correct them by altering the incoming beams. With the aid of adaptive optics, the spatial resolution of light-sheet microscopy can be improved by minimizing the detection defocus errors, a measurement of defocus aberration in the fluorescent detection, from 2.99 ± 1.34 μm to 0.06 ± 0.04 μm across the whole mouse embryo specimen [231]. More recently, multiple research groups combined two-photon and three-photon microscopy with adaptive optics, and the application in mouse brain imaging in vivo has seen substantial improvements in image quality at greater than 1 mm depth [232,233,234]. Recent advances in tissue clearing, which prevent unwanted light scattering by eliminating pigments and refractive index mismatch, have offered an alternative method for imaging through large and deep biological samples [235]. Ou et al. recently achieved optical transparency by applying tartrazine, a commonly used strong absorbing food color, to match the refractive index of the aqueous media [236].

Tracking with high background also remains challenging for both image-based methods and active feedback SVT methods. Signals from desired particles can easily be buried when the signal–background ratio is low. To achieve real-time 3D single-particle tracking in high-background conditions, Zhao et al. implemented lifetime gating together with two-photon excitation in a multi-detector active feedback tracking microscope to discriminate probe signals from background [237]. For active feedback fracking methods, the localization algorithm can also be improved to better distinguish particles from the background. Combined online Bayesian and windowed estimation of background and signal, developed by Niver et al., is a novel tracking algorithm that can estimate particle localization in the presence of a high (up to three times the particle intensity) and non-uniform background [161,238]. Combining the advances of microscopy methodology and algorithms, it is increasingly possible to achieve single-virus tracking in complex environments.

Future developments and studies of SVT in epithelium will provide solutions to unsolved puzzles in viral dynamics, such as the experimental demonstration of the “molecular walker” hypothesis of influenza viruses [239], and the mechanism behind viral penetration of the periciliary layer, which should block any particle larger than 40 nm in diameter [12]. Method development in the service of and knowledge gained from SVT in the epithelium will also bring insights into the dynamics of nanoscale objects over vast spatial and temporal scales, including inhalation-based drug/vaccine delivery, as well as reagent diffusion during chemical reactions [240,241].

## Data Availability

No new data were created or analyzed in this study. Data sharing is not applicable to this article.

## Abbreviations

2D	Two-dimensional
2P-LSM	Two-photon laser scanning microscope
3D	Three-dimensional
3D-FASTR	3D Fast Acquisition Scan by z-Translating Raster
3D-SMART	3D Single-Molecule Active Real-Time Tracking
3D-TrIm	3D tracking and imaging microscopy
APD	Single-photon counting avalanche photodiode
BS	Beam splitter
DLSM	Digital scanned light-sheet microscopy
EGFR	Epidermal growth factor receptor
EM-CCD	Electron multiplying charge-coupled device
EOD	Electro-optic deflector
ETL	Electrically tunable lens
FI	Fluorescence intermittency
FP	Fluorescent protein
FRET	Förster resonance energy transfer
GAGs	Glycosaminoglycans
HILO	Highly inclined and laminated optical sheet
HSV	Herpes simplex virus
IAV	Influenza A virus
iSCAT	Interferometric scattering microscopy
LDLR	Low-density lipoprotein receptor
LSCM	Laser scanning confocal microscopy
LSFM	Light-sheet fluorescence microscopy
MINFLUX	Minimal emission fluxes
mSPIM	Multidirectional selective plane illumination microscopy
NIR	Near-infrared
PAINT	Points accumulation for imaging in nanoscale topography
PCL	Periciliary layer
PS	Polystyrene
PSF	Point spread function
PVP	Pseudotyped viral particle
QD	Quantum dot
RESOLFT	Reversible saturable/switchable optical fluorescence transition
SA	Streptavidin
SARS-CoV-2	Severe acute respiratory syndrome coronavirus 2
SDCM	Spinning disk confocal microscopy
SPAD	Single-photon avalanche diode
SPT	Single-particle tracking
SVT	Single-virus tracking
TAG	Tunable acoustic gradient
TIRF	Total internal reflection fluorescence
TSUNAMI	Tracking single particles using nonlinear and multiplexed illumination
UCNPs	Upconversion nanoparticles
VLP	Virus-like particle
Vpr	Viral protein R
VSV	Vesicular stomatitis virus
